# The Preparation and After-Treatment of Section Cases

**Published:** 1905-06

**Authors:** 


**The Preparation and After-treatment of Section Cases.** By W. J. Stewart McKay, M.B., M.Ch., B.Sc. Pp. xx., 651. London: Baillière, Tindall & Cox. 1905.

On taking up this book the chief thought one has is that it is unduly lengthy, and inasmuch as the first 183 pages contain a great deal that can be found in any work on general surgery this seems to some extent unnecessary. The author's justification is partly to be found in the fact that he desires to treat in detail every circumstance connected with the subject-matter suggested by the title, and so far as this applies to the latter part of the book this object is praiseworthy, for it is evidently the outcome of much patient observation and considerable experience.

To those who are not very familiar with "section cases" the book is invaluable, for it deals with every conceivable circumstance likely to arise in connection therewith. The author's methods of treatment are clearly and minutely described in every part of the volume, and his clinical labours have resulted in a work which is absolutely reliable and highly praiseworthy.

In the treatment of diffuse peritonitis, about which there are so many opinions and much that is unknown, we did not notice any mention of massive saline injections, which appear to be valuable in many cases.

The book is heartily commended to all interested in abdominal surgery.

